# MMP-11 expression in early luminal breast cancer: associations with clinical, MRI, pathological characteristics, and disease-free survival

**DOI:** 10.1186/s12885-024-11998-0

**Published:** 2024-03-04

**Authors:** Sébastien Molière, Massimo Lodi, Suzanne Leblanc, Anne Gressel, Carole Mathelin, Fabien Alpy, Marie-Pierre Chenard, Catherine Tomasetto

**Affiliations:** 1 Institute of Genetics and Molecular and Cellular Biology, Illkirch, France; 2 Centre National de la Recherche Scientifique, UMR 7104, Illkirch, France; 3 Institut National de la Santé et de la Recherche Médicale, U1258, Illkirch, France; 4 University of Strasbourg, Illkirch, France; 5 Department of Radiology, Strasbourg University Hospital, Hôpital de Hautepierre, Strasbourg, France; 6 Breast and Thyroid Imaging Unit, ICANS, Strasbourg, France; 7 Groupe Rhénan de Pathologie, Schiltigheim, France; 8 Department of Senology, ICANS, Strasbourg, France; 9 Department of Pathology, Strasbourg University Hospital, Hôpital de Hautepierre, Avenue Molière, Strasbourg, France; 10 Department of Gynecology and Obstetrics, Strasbourg University Hospital, Hôpital de Hautepierre, Avenue Molière, Strasbourg, France

**Keywords:** Breast cancer, Microenvironment, MMP-11, Recurrence, Immunohistochemistry, Breast MRI

## Abstract

**Background:**

Early hormone-positive breast cancers typically have favorable outcomes, yet long-term surveillance is crucial due to the risk of late recurrences. While many studies associate MMP-11 expression with poor prognosis in breast cancer, few focus on early-stage cases. This study explores MMP-11 as an early prognostic marker in hormone-positive breast cancers.

**Methods:**

In this retrospective study, 228 women with early hormone-positive invasive ductal carcinoma, treated surgically between 2011 and 2016, were included. MMP-11 expression was measured by immunohistochemistry, and its association with clinical and MRI data was analyzed.

**Results:**

Among the patients (aged 31–89, median 60, with average tumor size of 15.7 mm), MMP-11 staining was observed in half of the cases. This positivity correlated with higher uPA levels and tumor grade but not with nodal status or size. Furthermore, MMP-11 positivity showed specific associations with MRI features. Over a follow-up period of 6.5 years, only 12 oncological events occurred. Disease-free survival was linked to Ki67 and MMP-11.

**Conclusion:**

MMP-11, primarily present in tumor-surrounding stromal cells, correlates with tumor grade and uPA levels. MMP-11 immunohistochemical score demonstrates a suggestive trend in association with disease-free survival, independent of Ki67 and other traditional prognostic factors. This highlights the potential of MMP-11 as a valuable marker in managing early hormone-positive breast cancer.

**Supplementary Information:**

The online version contains supplementary material available at 10.1186/s12885-024-11998-0.

## Introduction

Early invasive breast cancer, defined by tumors smaller than 2 cm and minimal or no lymph node involvement, has a prognosis strongly influenced by the tumor biological characteristics. While early-stage hormone-positive breast cancers have an excellent high 5-year survival rate [1, 2], still approximately 5% of these patients experience relapse, underscoring the need for long-term surveillance [3]. The Clinical Treatment Score at 5 years (CTS5) helps predict distant recurrence in ER-positive breast cancer patients [4], but its accuracy varies [5]. While gene profiling panels are effective for risk stratification [6, 7], their high cost limits their widespread use.

Matrix Metalloproteinase-11 (MMP-11) is a member of the Matrix Metalloproteinase (MMP) superfamily, a group of zinc-dependent endopeptidases known primarily for their capacity to degrade components of the extracellular matrix (ECM). MMP-11 is specifically expressed in cancer tissue, its presence in normal resting breast is undiscernible [8]. MMP-11 was first recognized for its elevated expression in invasive ductal carcinoma compared to in situ carcinoma, its presence in lobular carcinoma is low [9–11]. The correlation between MMP-11 and hormone receptor positivity remains a matter of debate [10–12].

Unlike many other MMPs, MMP-11 does not degrade major extracellular matrix components, but instead targets specific substrates, notably the insulin-like growth factor-binding protein-I (IGFBP-I) [13] suggesting a unique role in cancer progression. Moreover its negative regulation by MMP-14 suggests that MMP-11 might act within an MMP network [14].

Over the years, extensive studies have shed light on the prognostic significance of MMP-11 in breast cancer and other malignancies. Most studies concur that MMP-11 overexpression correlates with a poor prognosis in cancer cases. In particular, MMP-11 is one of a panel of 21 genes used to predict distant recurrence of breast cancer [6]. Preclinical studies, especially those involving mouse models, have emphasized its role in promoting early-stage breast cancer [15–17]. Additionally, MMP-11 function on stromal adipocytes near the tumor invasion front suggests a direct contribution to invasion [18]. However, the diversity in disease stages and variability in MMP-11 expression levels in previous studies necessitate further investigation. This study aims to explore the association between MMP-11and clinical, radiological, and pathological features of breast cancer and evaluate its potential as a prognostic marker in early-stage, hormone receptor-positive breast cancer, acknowledging the evolving landscape of breast cancer management and surveillance tools.

## Materials and methods

### Cell culture, transfection and western blot analysis

HEK 293T (CRL-3216) cell line was obtained from the American Type Culture Collection (ATCC). They were maintained in in Dulbecco’s modified Eagle’s medium supplemented with 10% (v/v) fetal bovine serum (FBS) and 1% penicillin-streptomycin. Plasmid transfections were done in P60 dishes using jetPEI® transfection reagent (Polyplus, France) and 3 µg of plasmids PQCXIP-MMP-11, PQCXIP-MMP-14, pCMV6-MMP2 and pCMV6-MMP9. Protein extracts were obtained by scraping in M-PER extraction buffer (Thermo Fisher Scientific, France) and 1X Complete protease inhibitor (Roche). For Western blot analysis, nearly equal amounts of proteins (20 µg) were separated on 8–18% SDS–PAGE and transferred onto nitrocellulose membranes. Membranes were blocked with milk 3% in 1× PBS, Tween-20 0.1%, and incubated overnight at 4 °C with anti-MMP-11 (4A9; 1/1000, IGBMC), anti-MMP-2 (5C3, 1/1000, IGBMC), anti-MMP-9 (4D2, 1/1000, IGBMC) and anti-Rab7 (#2576, 1/1000, IGBMC). Secondary horseradish peroxidase (HRP) conjugated anti-Mouse and anti-Rabbit antibodies were from Jackson ImmunoResearch. Signals were acquired using the (Amersham Imager 600).

### MMP-11 expression analyses from public databases

#### RNA expression of MMP-11 in single-cell studies

Transcriptional profiles of breast invasive carcinoma were obtained from a publicly available study encompassing 26 primary tumors, comprising 11 estrogen receptor-positive, 5 HER2-positive, and 10 triple-negative breast cancers. For a comprehensive description of the dataset, readers are referred to [19]. The data was accessed via the SingleCell Portal (singlecell.broadinstitute.org). The expression levels of MMP-11 were examined in all cell types present in the dataset. For fibroblasts, a deeper analysis was undertaken, MMP-11 expression was studied among the variety of fibroblast s. To identify variation in MMP-11 expression among the different cell types and fibroblast subtypes, pairwise comparisons were undertaken using a z-test. To account for multiple comparisons and reduce the probability of Type I error, *p*-values were adjusted using the Bonferroni correction method. In addition to the overall cellular landscape, MMP-11 expression was also evaluated in the context of specific breast cancer subtypes.

#### Correlation between protein and RNA expression

In our study, we analyzed the relationship between MMP-11 protein and RNA expression levels using proteogenomic data from The Cancer Genome Atlas (TCGA) project, which encompassed 30 primary breast tumors across three distinct centers. This data, accessible through the cBioPortal, provided us with z-scores for both mRNA and protein expressions of MMP-11. We employed Pearson’s linear regression analysis to determine the correlation between these two measures.

For this analysis, we utilized various Python libraries, including pandas (v.1.5.1) for data manipulation and analysis, scanpy (v.1.9.4) for handling large datasets of single-cell RNA sequencing data, seaborn (v.0.12.2) for data visualization, scipy.stats (v.1.11.2) for performing statistical tests, statsmodels (v.0.13.2) for estimating and interpreting models for statistical analysis, and matplotlib (v.3.5.1) for creating visualizations in Python.

### MMP-11 immunohistochemistry assay on early luminal breast cancer

#### Study design

We conducted a retrospective cohort study to assess MMP-11 expression in early breast cancer specimens obtained from women treated surgically as their primary intervention at Strasbourg University Hospital.

Eligibility criteria included:

1. Women aged > 18 diagnosed with primary infiltrating ductal carcinoma.
2. Treatment at Strasbourg University Hospital between January 2011 to December 2016.
3. Stage cT0-1 N0 and pT1N1a (tumor size no larger than 20 mm and either no lymph node involvement, or 1–2 involved lymph nodes).
4. Initial treatment consisted in surgery, without preceding neoadjuvant chemotherapy.
5. No biopsy during the 2 weeks preceding surgery.

Lobular carcinomas were excluded from this study, given that MMP-11 is poorly expressed in these tumors. Rare histological subtypes, such as metaplastic carcinomas, neuroendocrine tumors, adenoid cystic carcinomas, and tubular carcinomas were also excluded.

Patients initially presenting with metastatic disease or lacking available operative specimens were excluded.

#### Study endpoints

The primary objective was to identify, map and quantify MMP-11 expression in surgical specimens of breast tumors and correlate it with clinical, imaging and pathological markers. The secondary objective was to analyze event-free survival based on MMP-11 expression.

#### Biological Material

We sourced large tissue blocks fixed in formaldehyde and embedded in paraffin. Additionally, histological slides stained with hematoxylin-eosin were retrieved from the archives of the Pathology Department at Strasbourg University Hospital.

#### Clinical and Pathological Characteristics

Immunohistochemical methods were utilized to assess the expression of estrogen and progesterone receptors, following the guidelines set by the American Society of Clinical Oncology (ASCO) and the College of American Pathologists (CAP) [20, 21]. A hormone receptor status was deemed positive when the H-score was above 10. The Ki67 index was also determined through immunohistochemistry. Negativity of HER2 expression was confirmed by a dedicated immunohistochemical assay. Additional clinical and molecular characteristics included: (i) tumor grade, defined by the Elston and Ellis modified Scarff–Bloom–Richardson (SBR) grading system (Robbins et al. 1995), (ii) tumor stage, based on the TNM Classification from the American Joint Committee on Cancer (WHO Classification of Tumors Editorial Board 2019), (iii) tumor multifocality, (iv) presence of ductal carcinoma in situ, (v) determination of uPA (urokinase plasminogen activator) and PAI-1 (plasminogen activator inhibitor-1) level in tumor tissue by enzyme-linked immunoabsorbent assay (ELISA), as described in [22]. Survival duration was measured from the date of diagnosis. Patients were deemed lost to follow-up if there was no contact for over a year, and they hadn’t adhered to the recommended follow-up regimen.

#### MMP-11 immunohistochemistry technique

4 μm thick sections from paraffin blocks of formaldehyde-fixed breast tumors were placed on slides, dried at 56 °C for 1 h and then processed on a BenchMark ULTRA automated slide-stainer (Ventana). Except for the primary antibody, all used reagents were from Ventana. After deparaffinization and pretreatment (Cell Conditioning I for 30 min at 95 °C), sections were incubated with primary MMP-11 antibody, clone 5ST-4A9 (commercial reference: Sigma-Aldrich MABC1607-25UG) [23] at a concentration of 1/500 for 32 min at 37 °C. Immunoreactivity was detected with the iView DAB Detection Kit and counterstaining was performed with Hematoxylin II.

#### MMP-11 expression assessment

**Fig. 1:**
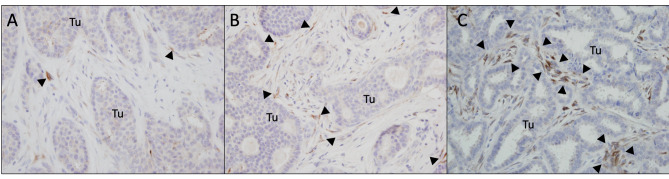
MMP-11 Immunohistochemistry. Assessment of MMP-11 expression density: **A**: less than one third of fibroblasts are stained (score 1), **B**: more than a third but less than two-third of the fibroblasts are stained (score 2), **C**: more than two-third of fibroblasts are stained (score 3). This 3-point score is then multiplied by the proportion of tumor stroma surface in which fibroblast-like MMP-11-positive cells are detected at low-magnification (0-100%), to obtain the MMP-11 immunohistochemical score (range 0-300). Tu: tumor cells islets, arrowheads: MMP-11 positive (cytoplasmic staining) fibroblast-like cells

MMP-11 expression was evaluated using a two-step approach [24]: (i) A visual estimation, at low magnification, of the proportion of tumor stroma surface in which fibroblast-like MMP-11-positive cells are detected (range 0 to 100%; e.g. a score of 20% indicates that MMP-11 expressing cells are detected in only part of the tumor stroma representing 20% of the total stromal surface). (ii) A semi-quantitative evaluation, at high magnification, of the ratio of MMP-11 positive to total number of fibroblast-like cells in MMP-11 positive stromal areas, scored as 0, 1, 2 and 3 (Fig.1). The MMP-11 immunohistochemical score was calculated by multiplying the results of the two assessments, resulting in a range of 0 to 300. Staining intensity was not considered in the evaluation, as this factor may fluctuate due to pre-analytic conditions. MMP-11 immunohistochemical score was carried out independently by two pathologists. Any disagreement was settled through consensus with a third reviewer.

#### MRI acquisition

All MRI scans were done on a 1.5T machine (MAGNETOM Aera, Siemens, Germany) and included at least the following sequences: T1-weighted images without fat suppression, T1-weighted images with fat suppression with and without gadolinium injection, T2- weighted images with or without fat suppression. T1w images of low quality, or those with significant artifacts, were omitted from the quantitative study. 2D axial T1-weighted images without fat suppression (TE: 12 ms, TR: 596 ms, flip angle: 150°slice thickness: 3 mm) were utilized for quantitative assessment of peritumor tissue.

#### MRI analysis

Breast MRI scans were evaluated for specific tumor characteristics, including BIRADS morphological descriptors [25], tumor necrosis presence, and the enhancement curve type. Additionally, breast density was rated on a 4-point scale based on the recommendations of the American College of Radiology (ACR) [25], and the area surrounding the tumor was quantitatively examined on one selected T1-weighted (T1w) image. These analytic steps encompassed: tumor segmentation, identification of the 5 mm-thick peritumoral region (pixels found in a tumor mask dilated by 5 mm, excluding those in the original tumor mask), image preprocessing, including intensity normalization, field correction and a comprehensive evaluation of the peritumoral region’s intensity histogram (Fig. 1). This histogram assessment covered metrics like mean/median intensity, intensity standard deviation, skewness, kurtosis, entropy, and histogram energy.

#### Survival analysis and determination of MMP-11 expression threshold

A 5-fold cross-validation framework was established. This approach involved dividing the dataset into five subsets, four for training and one for validation. For each training set, MMP11 expression thresholds were determined at different quantiles (33rd, 66th, and 90th). For survival analysis, the Kaplan-Meier survival method was used (log-rank test for comparisons). The optimal threshold for MMP11 expression was selected as the one that consistently showed the most significant difference (lowest *p*-value) in survival between the two groups across all validation sets.

#### Statistics

Univariate and multivariate analysis was conducted using linear regression models. To account for multiple comparisons in univariate analysis, *p*-values were adjusted using the Bonferroni correction method: a 2-tailed *p* value of < 0.0045 was therefore considered statistically significant. The analyses were done with the following Python libraries: pandas (v.1.5.1), statsmodels (v.0.13.2), lifelines (v.0.27.1), sklearn (v.1.3.2) and matplotlib (v.3.5.1).

## Results

### Immunohistochemical study of MMP-11 shows an expression in tumor stroma in about half of early luminal breast tumors

To assess if MMP-11 expression, detected through conventional IHC, could aid the management of early breast cancer patients, we examined its expression in a cohort of 228 women, with a median age of 60 years. Table 1 summarizes the clinical, pathological, and imaging characteristics of the cohort, with a repartition consistent with the known distribution of luminal cancers [26]. Of note, most tumors (86%) included in our study were classified as stage T1c, ranging between 1 and 2 cm.

**Table 1:** Characteristics of the population

	*N* = 228
Age	60.0 (31–89)
Tumor Size (mm)	15.7 (+/- 2.8, min–max 8–20)
**Number of involved lymph nodes**	
No involved node	160 (70%)
1–2 involved node	68 (30%)
Multifocality	84 (37%)
Extensive DCIS	94 (41%)
**Tumor grade (SBR)**	
Grade I	96 (42%)
Grade II	132 (58%)
**ER expression (H-score)**	277 (+/- 66)
ER-positive	224 (98%)
ER-negative	4 (2%)
**PR expression (H-score)**	205 (+/- 112)
PR-positive	191 (84%)
PR-negative	37 (16%)
**Ki67 expression (H-score)**	13.2 (+/- 8.4)
Ki67 < 15%	144 (63%)
Ki67 > 15%	84 (37%)
uPA level (ng/mg)	2.65 (+/- 2.2)
PAI1 level (ng/mg)	11.79 (+/- 11.87)
**MMP-11 expression (H-score)**	59 (+/- 84)
IH-MMP-11 < 50	138 (61%)
IH-MMP-11 > 50	90 (39%)
**Surgery type**	
Lumpectomy	167 (73%)
Mastectomy	61 (27%)
**Adjuvant therapy**	
Chemotherapy	73 (32%)
Radiation therapy	197 (84%)
Hormone therapy	228 (100%)

DCIS: ductal carcinoma in situ, ER: estrogen receptor, H-score: immunohistochemical score, PR: progesteron receptor, uPA: urokinase plasminogen activator, PAI-1: plasminogen activator inhibitor type-1, SBR: Scarff-Bloom-Richardson

**Fig. 2:**
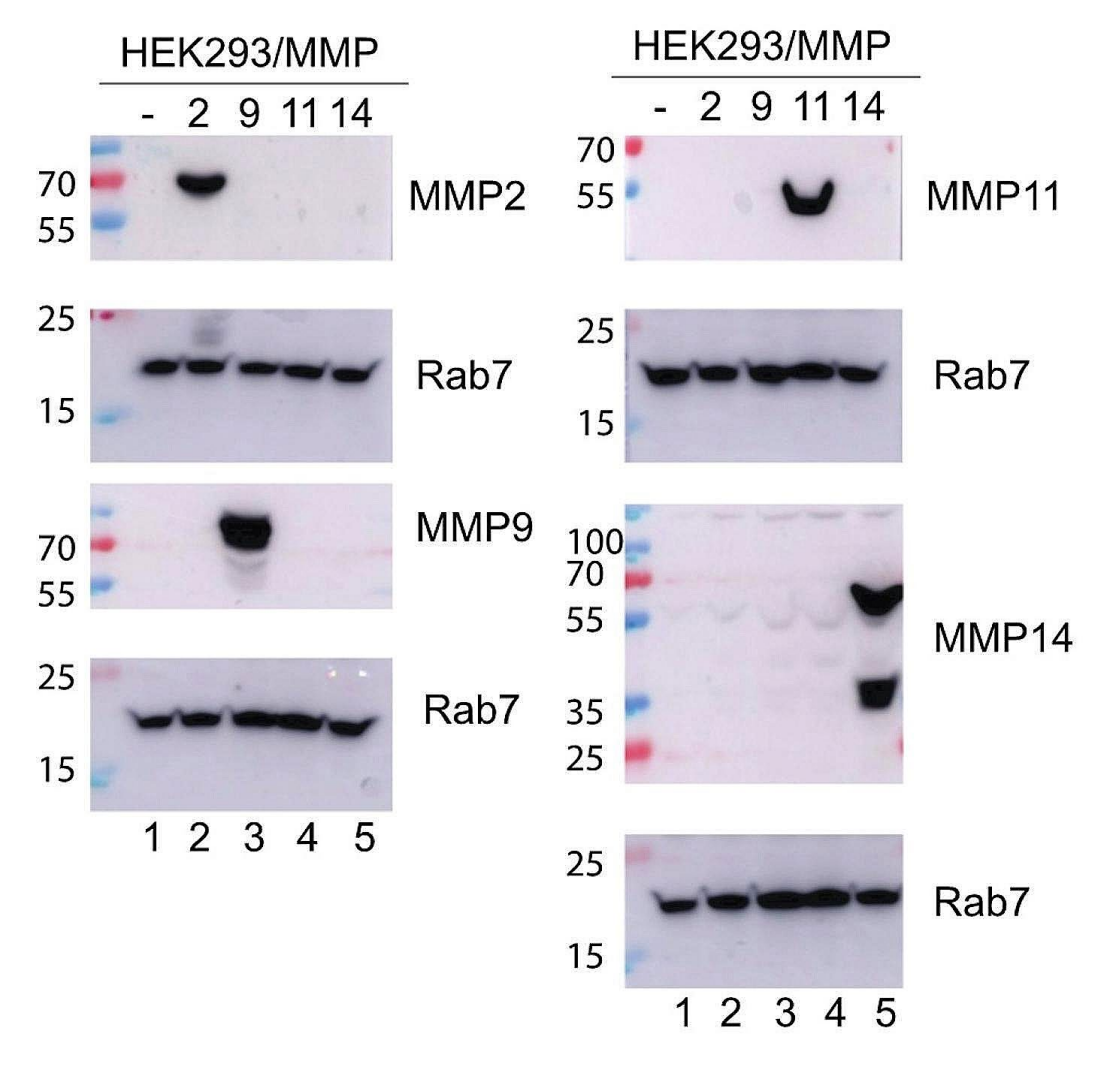
Characterization of the specificity of the anti MMP-11 antibody. Western blot analysis of different MMPs expression using the anti-MMP-11 antibody (5ST-4A9) in whole cell protein extracts (20 µg) of transfected HEK293 cells. Cells were either non-transfected (lane 1) or transfected with vectors encoding, MMP-2 (lane2), MMP-9 (lane 3), MMP-11 (lane 4) and MMP-14 (lane 5). The anti-MMP-11 specific antibody recognized a single protein of around 55KDa in MMP-11 transfected cells. Anti-MMP-2, MMP-9 and MMP-14 antibodies were utilized as controls and the anti-Rab7 antibody was used as a loading control

To verify the specificity of the anti-MMP-11 antibody, a series of transfections were performed in HeLa cells, with plasmids expressing MMP-2, MMP-9, MMP-14, and MMP-11 (Fig. 2). Western blot analysis confirmed the specificity of the 5ST4A9 anti-MMP-11 primary antibody, as none of the other MMPs tested were detected (Fig. 2). This result is consistent with the initial description of the anti-MMP-11 antibody [26] and confirm the its specificity for MMP-11.

**Fig. 3:**
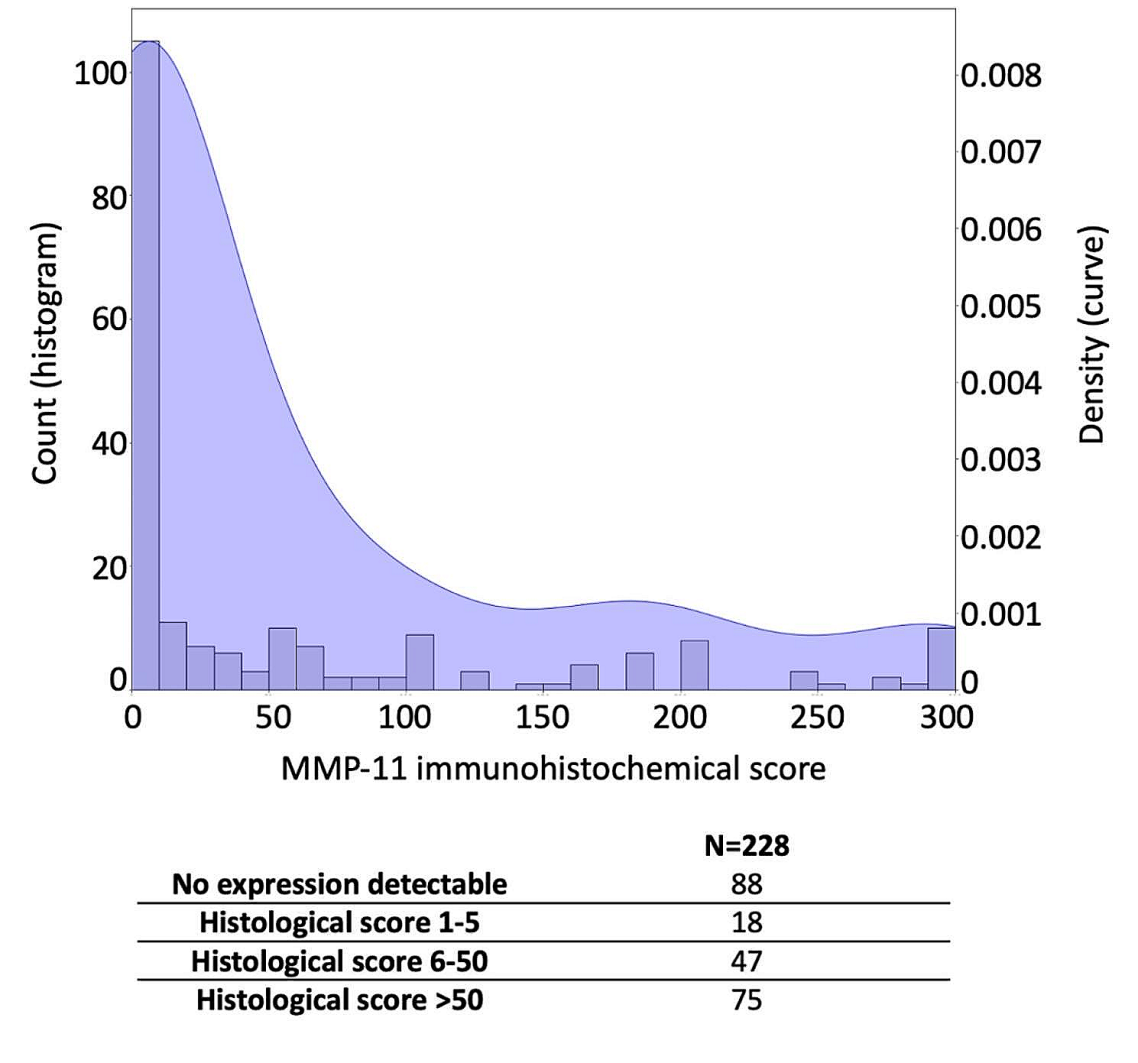
Distribution MMP-11 immunohistochemical score in the whole cohort, represented by histogram and density curve. Eighty-eight tumors exhibited no MMP-11 expression, 122/228 exhibited significant MMP-11 expression (HS > 5), and 75/228 exhibited strong MMP-11 expression (HS > 50). HS: immunohistochemical score

We next performed MMP-11 immunohistochemistry on the selected series of breast tumors (Fig. 1). This staining was done in parallel with the standard immunohistochemical analyses (ER, PR, HER2) performed in routine practice. A clear MMP-11 staining (score over 5) was observed in 53% of the cohort, as illustrated in Fig. 3. As illustrated in Fig. 1, MMP-11 staining was primarily observed in cells having a typical fibroblastic morphology interspersing tumor epithelial cells known as cancer-associated fibroblasts (CAFs). No significant staining of epithelial or inflammatory cells was observed.

### Association of MMP-11 expression with clinical and pathological features

Given that MMP11 is differentially expressed in this cohort of early breast cancer, we sought to determine its association with classical tumor characteristics and prognostic markers. In univariate analysis, only tumor grade and uPA level were associated with MMP-11 expression. This was confirmed with the multivariate linear regression model: MMP-11 immunohistochemical score was significantly associated with uPA (coefficient 8.8, *p* = 0.01) and tumor grade (coefficient 48.2, *p* = 0.007). Interestingly, MMP-11 immunohistochemical score did not correlate with tumor size, nodal status (Table 2). This analysis confirms that MMP-11 expression is independent of common stage-related prognostic factors.

**Table 2:** Regression multivariate analysis– clinical and pathological parameters associated with MMP-11 immunohistochemical score

	coefficient	*p*
Age	-0.7	0.3
Tumor Size (mm)	-1.3	0.6
Number of involved lymph nodes	-4.6	0.5
Multifocality	-25.1	0.1
Extensive DCIS	14.3	0.4
Tumor grade	48.2	**0.007**
Estrogen receptor (H-score)	-0.1	0.1
Progesteron receptor (H-score)	-0.02	0.9
Ki67	1.1	0.3
uPA level (ng/mg)	8.8	**0.01**
PAI1 level (ng/mg)	0.15	0.8

H-score: Immunohistochemical score

### In public dataset, MMP-11 is predominantly expressed in cancer-associated fibroblasts (CAFs)

**Fig. 4:**
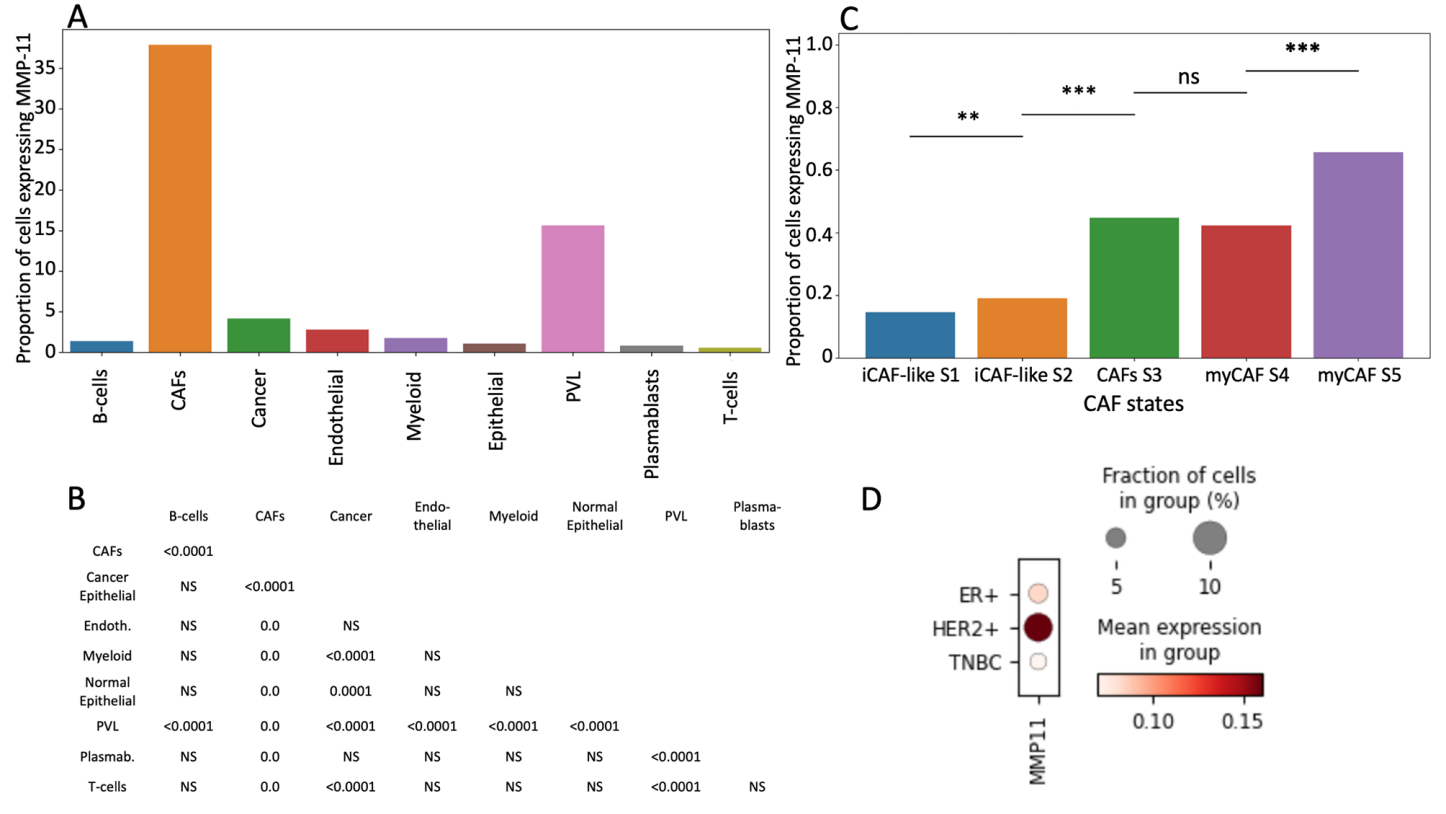
Analysis of MMP-11 expression across cell types, fibroblast subtypes, and breast cancer subtypes in primary tumors from public dataset. **A**. Proportion of cells expressing MMP-11 by cell type. **B**. Pairwise Comparisons of Cell Type Expressions: Please note that panel A shows the proportion of cells expressing MMP-11 for each cell type, and B displays the *p*-values of the multiple pairwise comparisons. **C**. Proportion of CAF Subtypes Expressing MMP-11. D. Dot-Plot of MMP-11 Expression by Tumor Subtypes. CAFs: cancer-associated fibroblasts, ER: estrogen receptor, PVL: perivascular-like cells, TNBC: triple negative breast cancer

Analysis of single-cell data from a large public dataset [19] (Fig. 4) revealed that MMP-11 is predominantly expressed in stromal cells, particularly in CAFs and perivascular cells. CAFs are heterogeneous and have been recently classified in different subtypes [27]. Among the different CAF subtypes, MMP-11 mRNA was detected in myCAF, a subpopulation enriched in myofibroblasts markers, much less in immunomodulating CAFs, and not in cancer cells. Of note, MMP-11 expression was detected in these CAFs across all three molecular subtypes of breast cancer, with a notable prevalence in HER2-positive tumors and, to a lesser extent, in estrogen receptor-positive cancers.

**Fig. 5:**
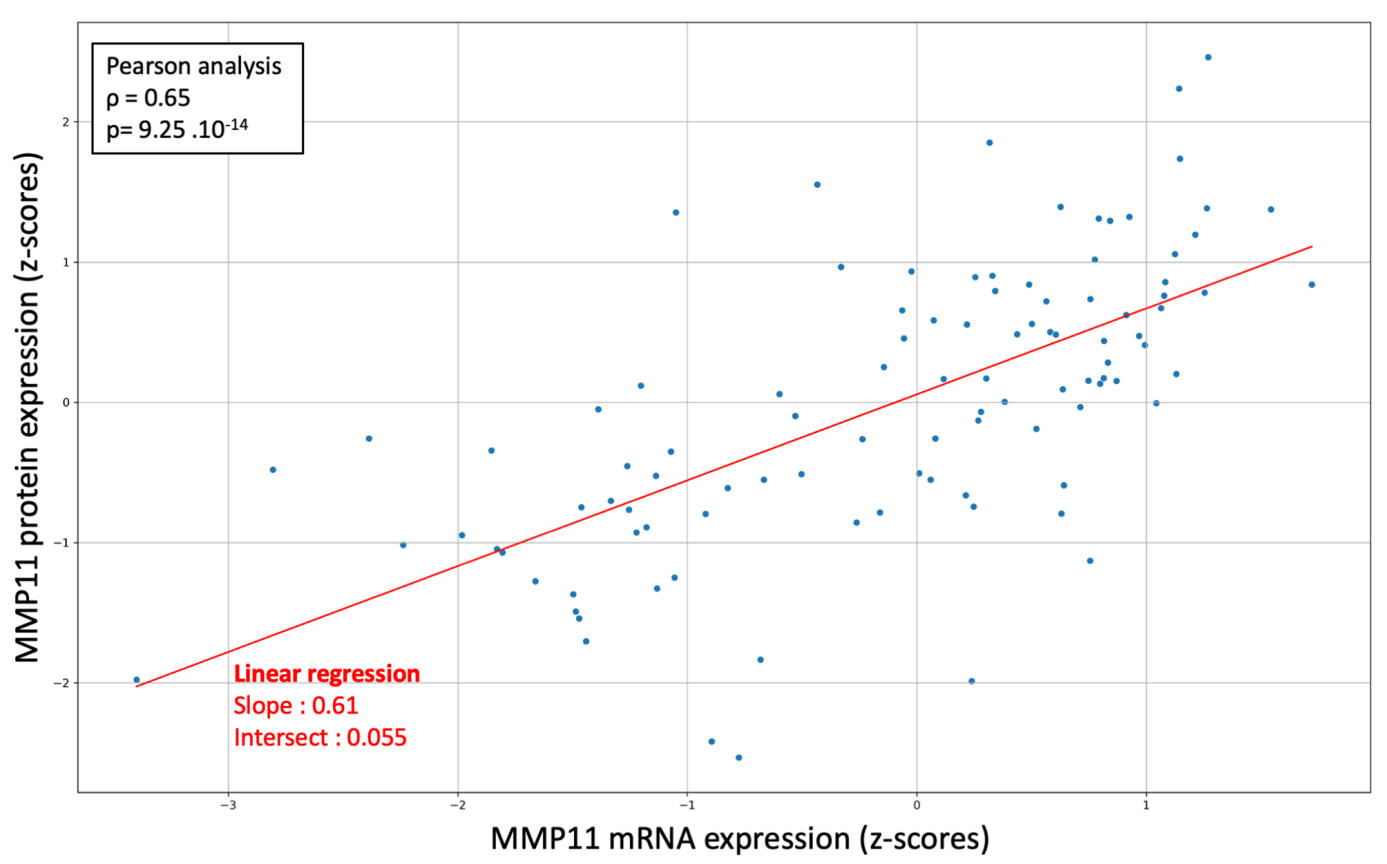
Correlation of MMP-11 protein and mRNA expression extracted from a public proteogenomic study from The Cancer Genome Atlas (TCGA) project (https://www.cancer.gov/tcga), using Pearson linear regression model. Protein and RNA expressions are expressed in z-score (relatively to the mean expression over the whole population)

Given that protein levels, as determined by immunohistochemistry, can substantially vary from RNA expression levels observed in single-cell studies, we conducted a supplementary proteogenomic analysis using data from The Cancer Genome Atlas (TCGA) project (https://www.cancer.gov/tcga). This analysis disclosed a statistically significant and moderately strong positive linear correlation between MMP-11 protein and mRNA expression levels (ρ = 0.65, *p* < 0.001, Fig. 5).

Altogether these results are consistent with our cohort study and with earlier studies. They support the notion that MMP-11 is specifically expressed in the tumor microenvironment.

### Association between MMP-11 immunohistochemical score and imaging features

**Fig. 6:**
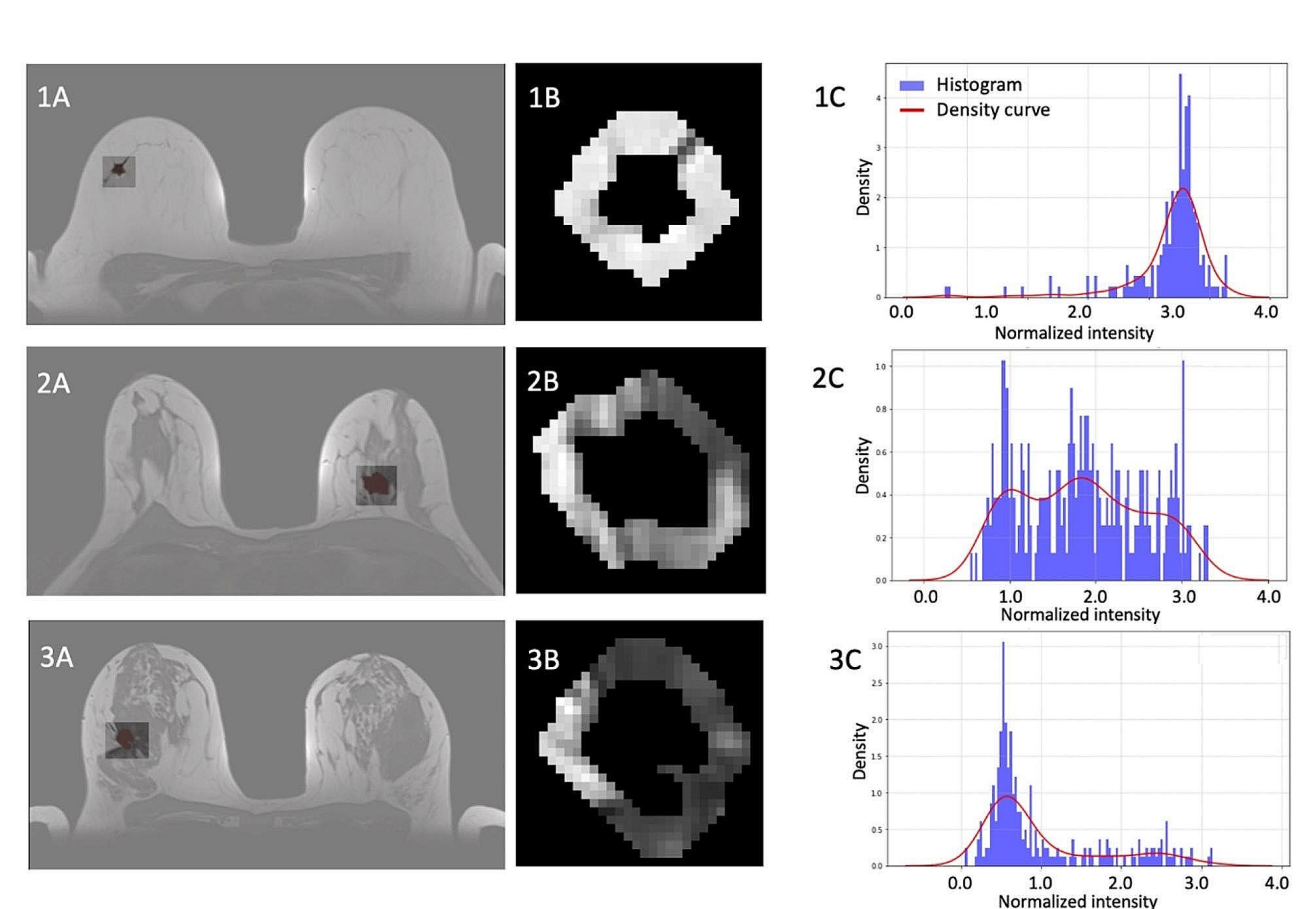
Quantitative analysis of the peri-tumor environment (pTE) in T1-weighted (T1w) magnetic resonance imaging. The panels display: **(A)** original T1w sequence with tumor segmentation overlaid for three cases labelled 1, 2 and 3, **(B)** corresponding segmentation of the 5 mm-thick outer margin, depicting the peritumoral region; and **(C)** a histogram representing the intensity distribution from the peritumoral region, enhanced with a superimposed density curve, for 3 patients with mostly fatty pTE (1), mixed fibroglandular and fatty pTE (2) and mostly fibroglandular pTE (3). The shape of the density curve and the position of the peaks vary in these 3 patients, depending on the relative proportion of fat (high intensity pixels) and fibroglandular tissue (low intensity pixels). pTE: peri-tumor environment, T1w: T1-weighted MRI

Dynamic contrast-enhanced breast magnetic resonance imaging (DCE-MRI) is a non-invasive imaging technique, increasingly utilized in preoperative scenarios. It not only identifies tumor margins, vascular patterns, and multifocality but also provides insights into the normal breast tissue and the surrounding peritumoral environment. We next wondered if MMP-11 expression could be associated with specific imaging features. To do so, we studied both tumor and peritumoral features on multiparametric MRI. All observed cancers appeared as small masses on MRI, with no indications of necrosis or oedema on T2-weighted imaging, an MRI technique where contrast depends on differences in the relaxation times of tissues, making fluid-filled structures appear bright. Furthermore, the gadolinium-enhancement curve type of the tumor exhibited no significant link to MMP-11 immunohistochemical score. We also found that breast density assessed semi-quantitatively [25] showed no correlation with MMP-11 expression in stromal cells. However, a thorough analysis of the peritumoral region’s fat-fibroglandular composition on T1-weighted imaging (Fig. 6), an MRI technique where contrast between different tissues is based on their specific T1 relaxation properties, revealed correlations between MMP-11 immunohistochemical score and several texture-related features: histogram entropy (coefficient 8.1, *p* = 0.02), histogram kurtosis (coefficient 25.4, *p* = 0.01), and histogram energy (coefficient 4.5, *p* = 0.06).

### Association between MMP-11 immunohistochemical score and disease-free survival

Over a median follow-up period of 76 months, 12 patients were lost to follow-up. During this time, 12 oncological events occurred: 3 local recurrences, 4 axillary recurrences, and 5 distant recurrences.

**Fig. 7:**
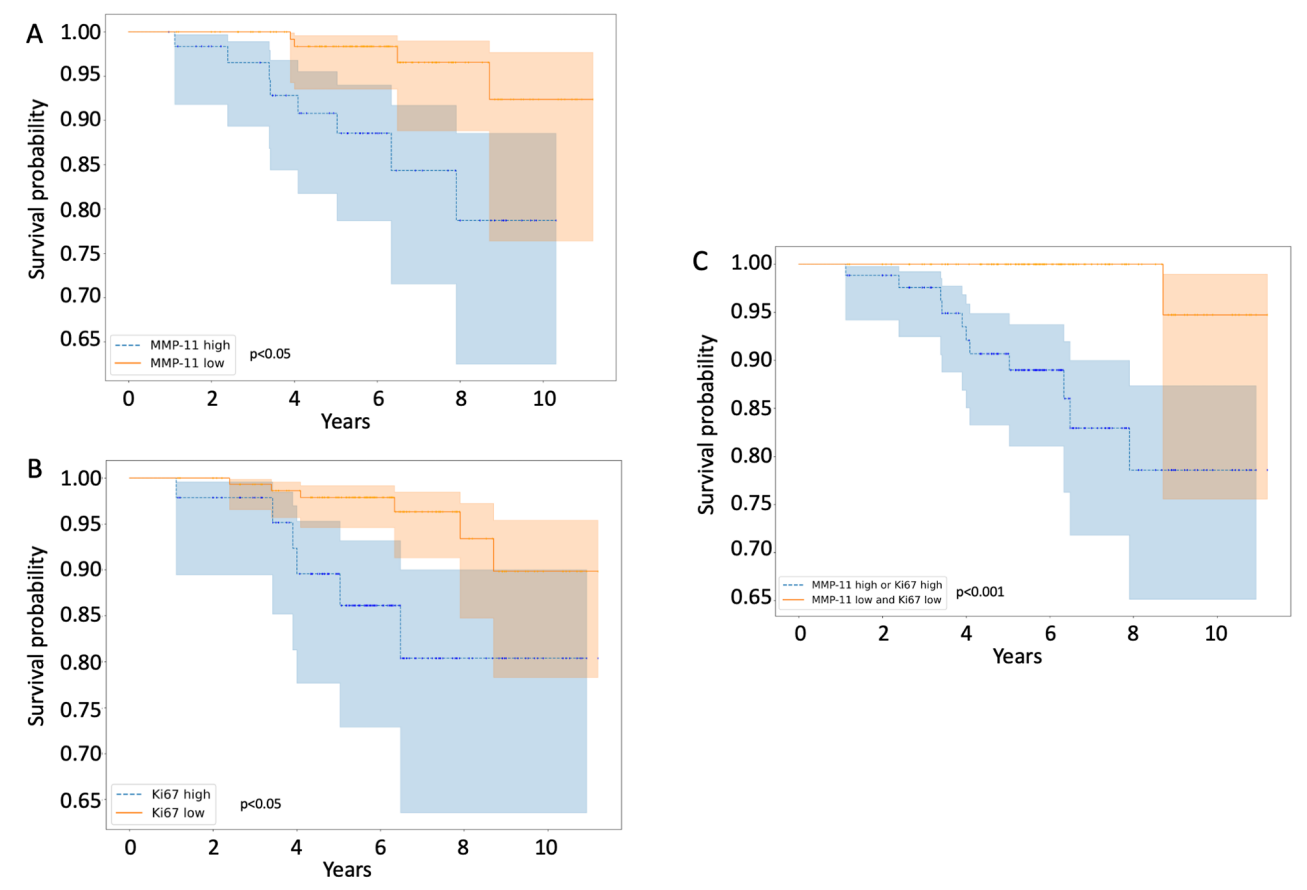
Disease-Free Survival Analysis for subgroups based on MMP-11 immunohistochemical score **(A)**, Ki67 score **(B)** and both scores **(C).** A illustrates the survival probabilities of two groups of women: those with a MMP-11 immunohistochemical score greater than 50 (blue dashed line) and those with a score 50 or below (orange solid line). B illustrates the survival probabilities of two groups of women: those with a Ki67 score greater than 15 (blue dashed line) and those with a score 15 or below (orange solid line). C illustrates the survival probabilities of two groups of women: those with either Ki67 score greater than 15% or MMP-11 immunohistochemical score greater than 50 (blue dashed line) and those considered “low-risk”, with Ki67 score lower than 15% and MMP-11 immunohistochemical score lower than 50 (orange solid line)

Using the Cox Proportional Hazards model, accounting for MMP-11 expression and other vital clinical and pathological prognostic factors, only two variables showed significant or near-significant associations with reduced DFS: elevated Ki67 (HR = 1.12, 95% CI [1.03–1.21], *p* = 0.01) and high MMP-11 expression (HR = 1.02, 95% CI [1.00-1.04], *p* = 0.05). A MMP-11 immunohistochemical score of 50 (66th percentile) was selected for survival analysis because it consistently showed the most significant difference in survival across all cross-validation sets. Figure 7A displays the Disease-Free Survival (DFS) curve for patients categorized by their MMP-11 immunohistochemical score, applying this cutoff. Regarding Ki67, the other key prognostic factor, a 15% cutoff demonstrated the most significant survival distinction and was thus utilized for patient stratification in the survival analysis, as shown in Fig. 7B. Ultimately, patients were divided into two groups: those with both low Ki67 and low MMP-11 expression, classified as low-risk, and those with elevated levels of either marker, as depicted in Fig. 7C. The combination of both markers was found to be a significant predictor of recurrence (HR = 2.80, 95% CI [0.72–4.82], *p* = 0.008).

This analysis shows the potential of a combined stratification using Ki67 and MMP-11 expression to predict the risk of recurrence in early luminal breast cancers.

## Discussion

In our investigation involving 228 women diagnosed with early-stage invasive ductal carcinoma of luminal types A and B, immunohistochemical analysis revealed that more than 50% of the tumors exhibited MMP-11 expression, predominantly within fibroblast-shaped cells present in the tumor stroma, known as cancer-associated fibroblasts (CAFs) [28].

A comprehensive review of clinical research on MMP-11 expression in human breast cancer, as summarized in Table 3, indicates a predominant reliance on immunohistochemistry for its detection. Comparatively, fewer studies have employed techniques such as northern blotting to assess RNA levels in tissue samples or in situ hybridization for detecting RNA on histological sections. Two studies [10, 29] performed both immunohistochemistry and in situ hybridization on a subset of cases, showing closely similar patterns of positivity. Exploiting data from publicly available databases, we observed a weak correlation between mRNA and protein levels for MMP-11. This disparity could be attributed to factors such as poor protein stability MMP-11 has a strong autoproteolytic activity [30] and is degraded by MMP-14 [14]. It is also important to note that while the detection of the protein itself is valuable, as MMP-11 is a proteolytic enzyme, the measurement of its enzymatic activity would provide a more direct assessment of its biological activity. However, currently, there is no robust system to make this kind of study.

**Table 3:** Previous studies of MMP-11 expression in human breast cancer

Type of MMP-11 analysis	Antibody used	Nb of BC cases	Population	Study design	Survival data	MMP-11 expression and localization	MMP-11 expression correlations	Year	Reference
NB	NA	92	92 primary BC 19 metastatic lymph nodes 91 normal tissue, 6 benign cases	Observational	no	Tumor tissue only	No association with stage, grade Positive association with ER+	1993	[55]
ISH	NA	68	65 primary BC 83% Invasive (IDC/ILC), 17% in situ Size 6-40 mm 59% ER-positive	Case-control	yes	82% of in situ BC 97% of invasive BC (IDC > ILC)	Positive association with grade and lower survival	1994	[9]
NB	NA	92	92 primary BC 89% invasive (IDC/ILC), 11% in situ N- and N+	Prospective cohort	yes	NA	No association with survival	1995	[11]
IHC	Monoclonal–In-house	111	111 primary BC 100% invasive (IDC/ILC) 80% T1-2 39% N-, 40% N+	Retrospective cohort	yes	76% of invasive BC (IDC > ILC) Fibroblast-like cells in tumor stroma only	Positive association with modified SBR grade and lower survival	1996	[24]
IHC ISH	Monoclonal–In-house	100	100 primary BC 20 benign cases 78% invasive (IDC/ILC, with 14% metaplastic) 28% in situ 96% T1-2 N- 37%, *N* + 63% (26% over 4 involved nodes)	Retrospective cohort	yes	80% of invasive BC 21% of in situ BC Fibroblast-like cells in tumor stroma only Epithelial tumor cells in metaplastic cancers	No association with grade, node statut, ER/PR Positive association with recurrence (univariate analysis only)	1998	[10]
ISH	NA	557	557 primary BC N- 35%, 65% N+ Tumor size 0.6-15 cm	Retrospective cohort	yes	89% of invasive BC Fibroblast-like cells in tumor stroma only	Positive association with younger age, higher grade, higher uPA Concomitant expression of cathepsin D, MMP-11 et uPA associated with lower survival	2001	[33]
IHC	Monoclonal–In-house	133	133 primary BC 100% invasive (IDC/ILC) n- 43%, *N* + 57%	Retrospective cohort	no	73% of invasive BC: -tumor stroma fibroblast-like cells: 65% (IDC > ILC) -epithelial tumor cells: 26%	Stromal expression positively associated with proliferation (TopoII α and Ki67) and decreased survival	2002	[34]
IHC	Monoclonal–LabVision Corporation, (Fremont, CA, USA)	124	124 primary BC 100% T1-2 48% N-, 52% N+	Case-control	yes	Fibroblast staining in 70% of invasive BC	Positive association with recurrence	2009	[31]
RT-PCR IHC (subset)	Monoclonal–Santa Cruz Biotechnology (CA, USA)	72	72 primary BC 75% T1-2, 25% T3-4 80% Stage I-II Paired with healthy ipsilateral breast tissue	Observational	no	Tumor tissue only	Positive association with lymph node involvement and high stage	2010	[29]
IHC (tissue arrays)	Monoclonal– LabVision Corporation (Fremont, CA, USA)	103	50 IDC (luminal 48%, T1-2, *N* + 58%) 23 ILC 14 mucinous 11 tubular / papillary 5 medullary	Observational	no	Tumour cells/fibroblast/MIC: 88/60/32% for IDC 100/91/79% for ILC 86/0/0% for mucinous 91/91/91% for tubular 100/100/100% of medullary	NA	2010	[32]
ISH	NA	30	30 ILC	Observational	no	53% of invasive lobular carcinoma Epithelial cells > stromal cells Staining pattern in epithelial cells different between invasive (pancytoplasmic) and non-invasive foci (beneath the plasma membrane)	NA	2011	[37]
IHC	Polyclonal- LabVision Corporation, (Fremont, CA, USA)	192	192 IDC 44% N-, 56% N+ 78% Stage I-II 56% Luminal A, 12% luminal B, 17% basal-like, 15% HER2 positive	Retrospective cohort	yes	80% of BC : epithelial tumor cells 20.8% of BC : tumor stroma fibroblast-like cells	Stromal expression positively associated with tumor size, high grade, tumor fibrosis, hormon-negative, HER2 positive, higher metastatic and recurrence rate No relevant association found for epithelial expression	2013	[12]
IHC (tissue arrays)	Monoclonal– LabVision Corporation (Fremont, CA, USA)	107	107 IDC 56% N-, 44% N+ 91% Stage I-II 43% hormone receptor positive	Observational	yes	Epithelial cancer cells: 87% (tumor center) and 97% (tumor front) Stromal cells: Fibroblasts 70% Mononucleate inflammatory cells 31%(center)-52%(invasive front)	MMP-11 expression by mononucleate inflammatory cells is associate with shorter relapse-free survival	2015	[50]

BC: breast cancer, DCIS: ductal carcinoma in situ, IDC: invasive ductal carcinoma, ILC: invasive lobular carcinoma, IHC: immunohistochemistry, ISH: in situ hybridation, MIC: mononuclear inflammatory cells, NB: northern blotting, N-: node negative, N+: node positive, SBR: Scarff Bloom Richardson

The number of MMP-11 positive cases found in this study is consistent with previous studies considering similar patient cohorts [31, 32]. The majority of past studies have identified MMP-11 predominantly within the tumor stroma, specifically in elongated, fibroblast-like cells located either intermingled with cancer cells in the center of the tumor or in the periphery in the invasive front [10, 12, 24, 31, 33, 34].In these studies MMP-11 staining was always intracellular, this can be explained by the rapid degradation of MMP11 in the extracellular space by an auto-degradation process or by other MMPs such as MMP14 [14, 30]. Our findings highlight the presence of MMP-11 in the tumor stroma, where it likely exerts its functional effects. Cancer-Associated Fibroblasts (CAFs) are increasingly recognized as key modulators of the tumor microenvironment of several types of solid tumor, including breast cancer. They exhibit vast molecular and genetic variations and are recruited from distinct sources. Some are native to the breast stroma, originating from resident tissue fibroblast or de-differentiated adipocytes, others may arise from the perivascular space during tumor evolution, or directly from tumor cells through epithelial-to-mesenchymal transition [35]. Distinct CAF subsets have been correlated with specific breast cancer molecular subtypes and prognostic value [36]. Although the classification of CAF subtypes has not reach a consensus yet among various studies, there is a general agreement on two main subtypes: myocontractile CAFs (myCAFs) that secrete extracellular matrix, and inflammatory CAFs (iCAFs), noted for their immunomodulating functions [27].

Intriguingly, several studies also reported MMP-11 expression within tumor cells themselves [10, 12, 32, 34, 37]. This expression pattern has been associated with certain breast cancer subtypes, like invasive lobular carcinoma [37] or metaplastic carcinoma of the breast [10], both tumor types characterized by an epithelial-to-mesenchymal transition (EMT) phenotype. In other studies, MMP-11 was found expressed in tumor cells of traditional invasive ductal carcinoma [12, 32, 34], though the epithelial expression was much less constantly associated with prognosis than the stromal expression. Similar findings have also emerged for other cancer types, including MMP-11 expression in spindle-shaped tumor cells of oral cavity cancers [38], in prostate and pancreatic tumor cells [39, 40]. In our study, we observed the absence of epithelial MMP-11 expression, which could be attributed to the specific composition of our cohort, notably the exclusion of lobular invasive carcinoma and rare subtypes. We also considered the potential cross-reactivity of the MMP-11 antibody with other MMPs, such as MMP2 (gelatinase A), MMP9 (gelatinase B), and MMP14 (membrane-type 1 MMP), which are commonly associated with tumor cells. This cross-reactivity may result in false-positive staining in epithelial cells. Analysis from a comprehensive single-cell database reinforced our findings, indicating that stromal cells, particularly CAFs and perivascular cells, are the predominant sources of MMP-11 expression.

Our research stands out as the only study to specifically concentrate on hormone receptor (HR)-positive breast cancers, the most common subtype. Previous reports either did not explicitly mention the proportion of HR-positive cases [9, 12, 24, 33, 34] or found them to constitute between 48% and 68% of their study populations [12, 32]. Interestingly, our analysis revealed a direct correlation between a high MMP-11 immunohistochemical score and an elevated tumor nuclear grade—a connection also supported by earlier studies that included a whole range of clinical and molecular breast cancer subtypes. Furthermore, parallels were drawn between overexpression of MMP-11 and increased markers of proliferation, such as Ki67 or TopoIIα [34]. Rapidly proliferating tumors necessitate enhanced metabolic support from their adjacent tissues. While MMPs are traditionally understood to remodel the extracellular matrix, the elevated expression of MMP-11 could also play a pivotal metabolic role. This could be mediated through a paracrine action on neighboring adipocytes, as indicated by previous studies [41–43]. Our study reinforces the idea that MMP-11 plays a pro-tumoral role in hormone-receptor positive breast cancers, the predominant subtype of breast cancers.

Our analysis identified a novel association of MMP-11 expression with specific peritumoral texture-related features on MRI, an aspect not explored in earlier studies. Breast MRI data analysis links peritumoral stroma characteristics with MMP-11 expression levels, suggesting a potential impact of MMP11 on peritumoral fat. The function of MMP-11 the adipose tissue and on adipocyte dedifferentiation has been documented experimentally in cells and animal studies [44]. This finding could open new avenues for non-invasive assessment of tumor biology using advanced imaging techniques, in particular MRI [45]. It is already known that MRI-detected peritumoral edema is associated with lymphovascular invasion, tumor necrosis and stromal fibrosis [46] and quantitative assessment of the peritumoral fat has been linked to cancer subtype [47].

The Urokinase plasminogen activator (uPA) plays a pivotal role in reshaping the extracellular matrix by converting plasminogen to plasmin, which subsequently activates various MMPs. While no direct molecular link between uPA and MMP-11 has been identified, their combined expression has been associated with an unfavorable prognosis [33]. The statistical association between the expression of these two proteases reinforces the idea that a matrix-associated protease activation cascade occurs in certain breast cancer types at an early stage.

Importantly, in terms of disease-free survival, our study noted that high MMP-11 expression, particularly when combined with a high Ki67 index, suggests an association with reduced DFS, even in the presence of a favorable overall prognosis, as demonstrated by the limited number of oncological events. This complements previous research using immunohistochemistry, such as [24] and [34], or RNA studies: for example, MMP-11 has been identified as part of a distinguishing genomic signature of “metastasis-associated fibroblasts” [48], a genomic signature related to breast cancer progression [49] and a genomic signature associated with the risk of recurrence of tamoxifen-treated, node-negative breast cancer [6]. Similarly, the study by Eiró et al. [50] further corroborates the importance of MMPs and TIMPs expressions within the fibroblastic compartment in stratifying prognostically significant microenvironment clusters. Ki67 was also identified as a significant prognostic factor in our study, where a 15% cutoff proved most effective for discrimination, aligning with findings from earlier research [51]. Notably, the combined analysis of Ki67 and MMP-11 in our cohort markedly improved prognostic accuracy. This was particularly evident as only one out of 125 patients with low expression levels of both Ki67 and MMP-11 experienced recurrence, compared to 11 out of 103 patients showing overexpression of either protein. These observations were recorded over a median follow-up period of 6.3 years.

Before implementing MMP-11 combined with Ki67 detection in the routine practice, external validation is needed in a prospective cohort. An online analysis of publicly accessible survival data from www.kmplot.com, encompassing 1496 hormone receptor-positive breast cancer patients selected based on endocrine therapy, reveals that higher RNA expressions of both Ki67 and MMP-11 are independently linked to increased recurrence risk in multivariate analysis. Specifically, higher Ki67 expression corresponds to a relative risk (RR) of 1.39 [95% CI: 1.11–1.73, *p* = 0.0042], while elevated MMP-11 expression is associated with a RR of 1.27 [95% CI: 1.01–1.58, *p* = 0.038]. Although these findings are based on RNA expression rather than protein levels, they still underscore the prognostic significance if MMP-11 in luminal breast cancer, suggesting of its potential as a biomarker, even when different methodologies are employed.

The management of breast cancer patient is constantly evolving: for HER2-enriched or high-grade triple-negative breast cancers, the administration of neoadjuvant or adjuvant systemic therapies is a common approach to reduce recurrence rates. In contrast, luminal tumors of similar size generally have a more favorable prognosis and some researchers suggest reconsidering the necessity of adjuvant chemotherapy [52] or even adjuvant endocrine therapy [53] in low-risk patients. Tumor size continues to be a critical factor, which is why our study predominantly focused on T1 tumors. A significant portion of the tumors included in our analysis fell within the 1 to 2 cm size range, mirroring the populations observed in other research dedicated to de-escalation strategies [52]. This stratification approach agrees with the broader goal of personalized medicine in oncology, where treatment decisions are guided by specific biomarkers.

Given its relative simplicity and low cost, immunohistochemical evaluation of MMP-11 expression in tumor tissues offers a complementary prognostication tool, allowing for a more nuanced risk assessment, tailored follow-up of these early, hormone receptors positive, breast tumors.

Our study has some limitations. Despite being one of the more extensive clinical studies on this topic, the limited number of oncological events may hamper its power to highlight significant statistical differences. Additionally, the immunohistochemical assessment’s semi-quantitative nature, though straightforward, can introduce potential inter-reader variability and might be affected by technical inconsistencies. This study did not primarily focus on tumor heterogeneity, but it is important to recognize that a low MMP-11 immunohistochemical score can be associated with localized MMP-11 expression, which might indicate local progression, through MMP-11 function on substrates like IGFBP1 and Collagen VI [13, 54]. However, it is worth noting that currently, we lack a straightforward method to concurrently assess the presence of MMP11 and its substrates in tumors. Eventually, given the lack of specific fibroblast marker, we relied on cell morphology to identify CAFs, which is a common issue when studying fibroblasts [27].

## Conclusion

In conclusion, our study in early luminal breast cancer patients reveals that MMP-11 expression, predominantly seen in cancer-associated fibroblasts, is linked with tumor grade and uPA levels. Both Ki67 and MMP-11 expressions are indicators of disease-free survival. Of interest, their combined assessment facilitates stratification of patients into low-risk and high-risk groups. The high-risk group exhibits a notably increased risk of recurrence. This highlights the potential of these markers in guiding personalized treatment strategies.

### Electronic supplementary material

Below is the link to the electronic supplementary material.

Supplementary Material 1. Title of data.: Characterization of the specificity of the anti MMP-11 antibody by Western blot (uncropped blots). Description of data: Western blot analysis of different MMPs expression using the anti-MMP-11 antibody in whole cell protein extracts (20 µg) of transfected HEK293 cells. Cells were either transfected with vectors encoding, MMP-2 (A), MMP-9 (B), MMP-14 (C) and MMP-11 (D). GAPDH/Rab7 was used as a loading control (E).

## Data Availability

The data presented in this study are available on request through the corresponding author.

## Abbreviations

BC: breast cancer
DCIS: ductal carcinoma in situ
DFS: disease-free survival
ER: estrogen receptor
HER2: Human epidermal growth factor receptor-2
IDC: invasive ductal carcinoma
ILC: invasive lobular carcinoma
IHC: immunohistochemistry
ISH: in situ hybridation
PAI-1: plasminogen activator inhibitor type-1
PR: progesteron receptor
RR: relative risk
SBR: Scarff Bloom Richardson
uPA: urokinase plasminogen activator
