# Supplementary figures and images for: MMP-11 expression in early luminal breast cancer: associations with clinical, MRI, pathological characteristics, and disease-free survival
Source: BMC Cancer. 2024 Mar 4;24:295. doi: 10.1186/s12885-024-11998-0 (PMC10913243; doi:10.1186/s12885-024-11998-0)

A

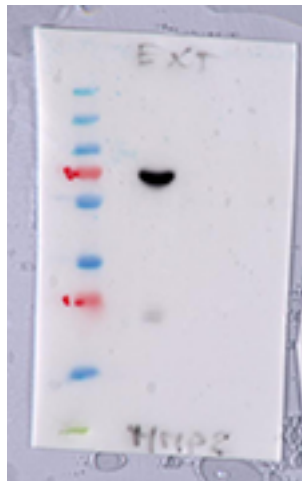

probed MMP2

B

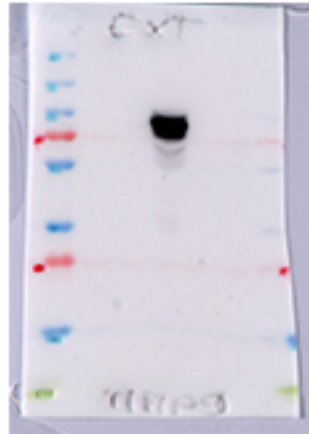

probed MMP9

C

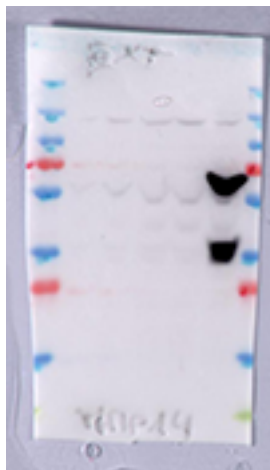

probed MMP14

D

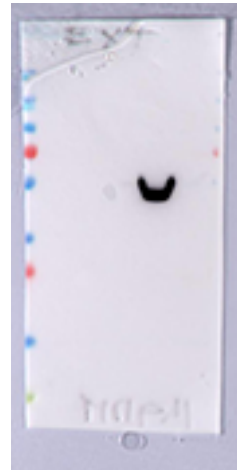

probed MMP11

E

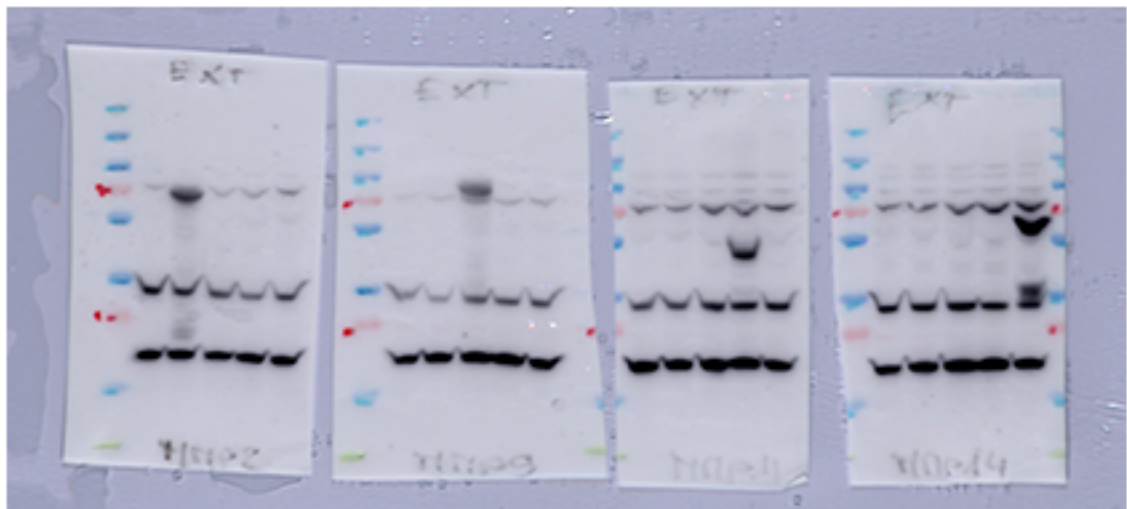

reprobed GAPDH/ reprobed RAB7

Supplement: Supplementary file 1 — Supplementary Material 1. Title of data.: Characterization of the specificity of the anti MMP-11 antibody by Western blot (uncropped blots). Description of data: Western blot analysis of different MMPs expression using the anti-MMP-11 antibody in whole cell protein extracts (20 µg) of transfected HEK293 cells. Cells were either transfected with vectors encoding, MMP-2 (A), MMP-9 (B), MMP-14 (C) and MMP-11 (D). GAPDH/Rab7 was used as a loading control (E). [file 12885_2024_11998_MOESM1_ESM.pdf]
